# Dichlorido[2,2′-(oxydimethyl­ene)dipyridine]copper(II)

**DOI:** 10.1107/S1600536808034612

**Published:** 2008-10-31

**Authors:** Jin Min Li

**Affiliations:** a Chemistry and Chemical Engineering College, Shanxi Datong University, Datong 037009, People’s Republic of China

## Abstract

In the title complex, [CuCl_2_(C_12_H_12_N_2_O)], the Cu^II^ ion is coordinated in a distorted trigonal-bipyramidal environment. In the crystal structure, there is a weak π–π stacking inter­action between symmetry-related pyridine rings, with a centroid-to-centroid distance of 3.8134 (17) Å. In addition, there is relatively close contact between the pyridine ring π-system and a symmetry-related Cu^II^ ion (Cu⋯centroid distance of 3.868 Å).

## Related literature

For the isotypic Cd and Zn analogs of the title compound, see: Li (2007 ▶) and Li (2008 ▶), respectively.
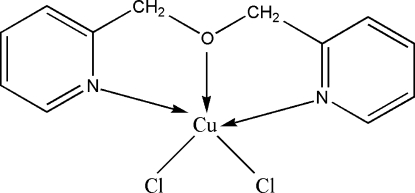


## Experimental

### 

#### Crystal data

[CuCl_2_(C_12_H_12_N_2_O)]
*M* *_r_* = 334.68
Monoclinic, 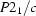

*a* = 8.1599 (10) Å
*b* = 12.5534 (15) Å
*c* = 15.3846 (14) Å
β = 123.574 (9)°
*V* = 1313.0 (3) Å^3^
*Z* = 4
Mo *K*α radiation
μ = 2.06 mm^−1^
*T* = 298 (2) K
0.46 × 0.40 × 0.34 mm

#### Data collection

Bruker SMART APEX CCD diffractometer
Absorption correction: multi-scan (*SADABS*; Sheldrick, 1996 ▶) *T*_min_ = 0.451, *T*_max_ = 0.541 (expected range = 0.414–0.497)
5381 measured reflections
2323 independent reflections
2152 reflections with *I* > 2σ(*I*)
*R*_int_ = 0.016

#### Refinement

*R*[*F* ^2^ > 2σ(*F* ^2^)] = 0.024
*wR*(*F* ^2^) = 0.066
*S* = 1.09
2323 reflections
163 parameters
H-atom parameters constrained
Δρ_max_ = 0.33 e Å^−3^
Δρ_min_ = −0.27 e Å^−3^

### 

Data collection: *SMART* (Bruker, 2007 ▶); cell refinement: *SAINT* (Bruker, 2007 ▶); data reduction: *SAINT*; program(s) used to solve structure: *SHELXTL* (Sheldrick, 2008 ▶); program(s) used to refine structure: *SHELXTL*; molecular graphics: *SHELXTL*; software used to prepare material for publication: *SHELXTL*.

## Supplementary Material

Crystal structure: contains datablocks I, global. DOI: 10.1107/S1600536808034612/lh2712sup1.cif

Structure factors: contains datablocks I. DOI: 10.1107/S1600536808034612/lh2712Isup2.hkl

Additional supplementary materials:  crystallographic information; 3D view; checkCIF report

## Figures and Tables

**Table:** 

Cl1—Cu1	2.4109 (6)
Cl2—Cu1	2.2538 (6)
Cu1—N2	2.0021 (17)
Cu1—N1	2.0092 (18)
Cu1—O1	2.0813 (14)

**Table:** 

N2—Cu1—N1	155.06 (7)
N2—Cu1—O1	78.29 (6)
N1—Cu1—O1	77.99 (7)
N2—Cu1—Cl2	98.17 (5)
N1—Cu1—Cl2	97.71 (6)
O1—Cu1—Cl2	147.65 (4)
N2—Cu1—Cl1	93.36 (5)
N1—Cu1—Cl1	96.97 (6)
O1—Cu1—Cl1	96.81 (4)
Cl2—Cu1—Cl1	115.53 (2)

**Fig. 1.**
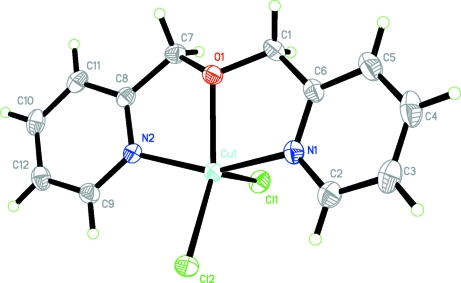
View of complex (I), showing the the atom numbering scheme with thermal ellipsoids drawn at the 30% probability level

## supplementary crystallographic information

### Comment

2,2'-[oxydi(methylene)]dipyridine is an useful tridentate terminal ligand and the Cd^II^ complex with it as ligand has already been published (Li, 2007). Herein the crystal structure of the title complex, (I), is reported.

The molecular structure of (I) is shown in Fig. 1. The atom Cu1 is coordinated in a distorted trigonal-bipyramidal environment (Table 1). In the crystal structure, there is a weak π-π stacking interaction between symmetry related pyridyl rings, with the relevant distances being *Cg*1···*Cg*1^i^ = 3.8134 (17) Å and a perpendicular distance of 3.556 Å [symmetry code (i) -*x*, 2 - *y*, -*z*; *Cg*1 is the centroid of the N1/C2—C6 ring]. In addition, there is close contact between a π-ring system and symmetry related Cu atom, with the relative distances being: *Cg*2···Cu1^ii^ = 3.868 Å, *Cg*2_perp_···Cu1^ii^ = 3.635 Å [symmetry code: (ii) -*x*, 1 - *y*, -*z*; *Cg*2 is the centroid of the N2/C8—C12 ring; *Cg*2_perp_···Cu1^ii^ is the perpendicular distance from Cu1^ii^ to N2/C8—C12 ring]. The title compound is isostructural with the Cadmium analog (Li, 2007) although the Cd analog was solved and refined in the non-standard *P*2_1_/*n* setting of space group *P*2_1_/*c*.

### Experimental

6 ml me thanol solution of 2,2'-[oxydi(methylene)]dipyridine (0.0418 g, 0.209 mmol) was added into 8 ml H_2_O solution containing CuCl_2_.2H_2_O (0.0362 g, 0.212 mmol), and the mixed soulution was stirred for a few minutes. The green single crystals were obtained after the solution had been allowed to stand at room temperature for two weeks.

### Refinement

All H atoms were placed in calculated positions and refined as riding, C—H = 0.93–0.97 Å, with *U*_iso_(H) = 1.2*U*_eq_(C).

### Crystal data

**Table:**

[CuCl_2_(C_12_H_12_N_2_O)]	*F*(000) = 676
*M**_r_* = 334.68	*D*_x_ = 1.693 Mg m^−^^3^
Monoclinic, *P*2_1_/*c*	Mo *K*α radiation, λ = 0.71073 Å
Hall symbol: -P 2ybc	Cell parameters from 4907 reflections
*a* = 8.1599 (10) Å	θ = 2.3–28.3°
*b* = 12.5534 (15) Å	µ = 2.06 mm^−^^1^
*c* = 15.3846 (14) Å	*T* = 298 K
β = 123.574 (9)°	Block, green
*V* = 1313.0 (3) Å^3^	0.46 × 0.40 × 0.34 mm
*Z* = 4	

### Data collection

**Table:**

Bruker SMART APEX CCD diffractometer	2323 independent reflections
Radiation source: fine-focus sealed tube	2152 reflections with *I* > 2σ(*I*)
graphite	*R*_int_ = 0.016
φ and ω scans	θ_max_ = 25.0°, θ_min_ = 2.3°
Absorption correction: multi-scan (SADABS; Sheldrick, 1996)	*h* = −9→9
*T*_min_ = 0.451, *T*_max_ = 0.541	*k* = −11→14
5381 measured reflections	*l* = −18→13

### Refinement

**Table:**

Refinement on *F*^2^	Primary atom site location: structure-invariant direct methods
Least-squares matrix: full	Secondary atom site location: difference Fourier map
*R*[*F*^2^ > 2σ(*F*^2^)] = 0.024	Hydrogen site location: inferred from neighbouring sites
*wR*(*F*^2^) = 0.066	H-atom parameters constrained
*S* = 1.09	*w* = 1/[σ^2^(*F*_o_^2^) + (0.0361*P*)^2^ + 0.5176*P*] where *P* = (*F*_o_^2^ + 2*F*_c_^2^)/3
2323 reflections	(Δ/σ)_max_ = 0.001
163 parameters	Δρ_max_ = 0.33 e Å^−^^3^
0 restraints	Δρ_min_ = −0.27 e Å^−^^3^

### Special details

**Table:**

Geometry. All e.s.d.'s (except the e.s.d. in the dihedral angle between two l.s. planes) are estimated using the full covariance matrix. The cell e.s.d.'s are taken into account individually in the estimation of e.s.d.'s in distances, angles and torsion angles; correlations between e.s.d.'s in cell parameters are only used when they are defined by crystal symmetry. An approximate (isotropic) treatment of cell e.s.d.'s is used for estimating e.s.d.'s involving l.s. planes.
Refinement. Refinement of *F*^2^ against ALL reflections. The weighted *R*-factor *wR* and goodness of fit *S* are based on *F*^2^, conventional *R*-factors *R* are based on *F*, with *F* set to zero for negative *F*^2^. The threshold expression of *F*^2^ > σ(*F*^2^) is used only for calculating *R*-factors(gt) *etc*. and is not relevant to the choice of reflections for refinement. *R*-factors based on *F*^2^ are statistically about twice as large as those based on *F*, and *R*- factors based on ALL data will be even larger.

### Fractional atomic coordinates and isotropic or equivalent isotropic displacement parameters (Å^2^)

**Table:**

	*x*	*y*	*z*	*U*_iso_*/*U*_eq_	
C1	−0.0360 (3)	0.76246 (18)	−0.10853 (17)	0.0407 (5)	
H1A	−0.1499	0.7554	−0.1795	0.049*	
H1B	0.0687	0.7967	−0.1103	0.049*	
C2	−0.0341 (4)	0.8555 (2)	0.1167 (2)	0.0481 (6)	
H2	0.0297	0.8372	0.1868	0.058*	
C3	−0.1666 (4)	0.9378 (2)	0.0794 (2)	0.0576 (7)	
H3	−0.1909	0.9750	0.1235	0.069*	
C4	−0.2628 (4)	0.9644 (2)	−0.0242 (2)	0.0604 (7)	
H4	−0.3532	1.0200	−0.0516	0.072*	
C5	−0.2232 (4)	0.9072 (2)	−0.0868 (2)	0.0507 (6)	
H5	−0.2882	0.9231	−0.1574	0.061*	
C6	−0.0864 (3)	0.82611 (17)	−0.04415 (17)	0.0357 (5)	
C7	0.1210 (3)	0.59462 (18)	−0.09417 (16)	0.0369 (5)	
H7A	0.2093	0.6368	−0.1039	0.044*	
H7B	0.0243	0.5614	−0.1601	0.044*	
C8	0.2343 (3)	0.51119 (17)	−0.01249 (15)	0.0320 (4)	
C9	0.3812 (3)	0.46002 (18)	0.15917 (17)	0.0368 (5)	
H9	0.4090	0.4735	0.2255	0.044*	
C10	0.4063 (3)	0.34591 (18)	0.0442 (2)	0.0427 (5)	
H10	0.4507	0.2836	0.0311	0.051*	
C11	0.2964 (3)	0.41864 (18)	−0.03445 (18)	0.0405 (5)	
H11	0.2645	0.4055	−0.1016	0.049*	
C12	0.4491 (3)	0.36715 (19)	0.14218 (19)	0.0420 (5)	
H12	0.5231	0.3193	0.1965	0.050*	
Cl1	0.46540 (8)	0.76638 (5)	0.12142 (4)	0.04202 (15)	
Cl2	0.20944 (9)	0.63873 (5)	0.25169 (4)	0.04202 (15)	
Cu1	0.18810 (4)	0.673873 (19)	0.102078 (18)	0.03121 (10)	
N1	0.0076 (3)	0.80023 (14)	0.05709 (14)	0.0359 (4)	
N2	0.2764 (2)	0.53190 (13)	0.08332 (13)	0.0310 (4)	
O1	0.0261 (2)	0.66050 (11)	−0.05971 (11)	0.0324 (3)	

### Atomic displacement parameters (Å^2^)

**Table:**

	*U*^11^	*U*^22^	*U*^33^	*U*^12^	*U*^13^	*U*^23^
C1	0.0432 (12)	0.0411 (13)	0.0352 (11)	0.0067 (10)	0.0201 (10)	0.0108 (10)
C2	0.0580 (15)	0.0417 (13)	0.0490 (14)	0.0070 (12)	0.0323 (12)	−0.0021 (11)
C3	0.0632 (17)	0.0426 (14)	0.0748 (19)	0.0087 (13)	0.0430 (16)	−0.0074 (13)
C4	0.0536 (15)	0.0377 (14)	0.086 (2)	0.0142 (12)	0.0366 (15)	0.0069 (14)
C5	0.0452 (13)	0.0427 (14)	0.0552 (15)	0.0097 (11)	0.0221 (12)	0.0123 (12)
C6	0.0320 (11)	0.0316 (11)	0.0395 (12)	0.0005 (8)	0.0173 (10)	0.0059 (9)
C7	0.0425 (12)	0.0396 (12)	0.0326 (11)	−0.0007 (10)	0.0232 (10)	−0.0024 (9)
C8	0.0300 (10)	0.0321 (11)	0.0352 (10)	−0.0050 (8)	0.0189 (9)	−0.0044 (9)
C9	0.0341 (11)	0.0374 (12)	0.0345 (11)	0.0015 (9)	0.0162 (9)	0.0023 (9)
C10	0.0391 (12)	0.0308 (11)	0.0646 (16)	0.0000 (9)	0.0327 (12)	−0.0045 (11)
C11	0.0420 (12)	0.0394 (13)	0.0470 (13)	−0.0066 (10)	0.0290 (11)	−0.0112 (10)
C12	0.0355 (11)	0.0354 (12)	0.0523 (14)	0.0047 (10)	0.0225 (11)	0.0072 (10)
Cl1	0.0417 (3)	0.0411 (3)	0.0482 (3)	−0.0068 (2)	0.0279 (3)	−0.0045 (2)
Cl2	0.0550 (3)	0.0420 (3)	0.0353 (3)	−0.0009 (3)	0.0289 (3)	0.0015 (2)
Cu1	0.03650 (16)	0.02880 (16)	0.02902 (15)	0.00285 (10)	0.01855 (12)	0.00079 (9)
N1	0.0363 (10)	0.0307 (9)	0.0402 (10)	0.0028 (8)	0.0209 (8)	0.0014 (8)
N2	0.0314 (8)	0.0288 (9)	0.0325 (9)	−0.0017 (7)	0.0175 (7)	−0.0023 (7)
O1	0.0341 (8)	0.0336 (8)	0.0286 (7)	0.0023 (6)	0.0168 (6)	0.0025 (6)

### Geometric parameters (Å, °)

**Table:**

C1—O1	1.428 (3)	C7—H7B	0.9700
C1—C6	1.496 (3)	C8—N2	1.341 (3)
C1—H1A	0.9700	C8—C11	1.382 (3)
C1—H1B	0.9700	C9—N2	1.343 (3)
C2—N1	1.336 (3)	C9—C12	1.376 (3)
C2—C3	1.372 (4)	C9—H9	0.9300
C2—H2	0.9300	C10—C12	1.372 (4)
C3—C4	1.373 (4)	C10—C11	1.379 (3)
C3—H3	0.9300	C10—H10	0.9300
C4—C5	1.375 (4)	C11—H11	0.9300
C4—H4	0.9300	C12—H12	0.9300
C5—C6	1.379 (3)	Cl1—Cu1	2.4109 (6)
C5—H5	0.9300	Cl2—Cu1	2.2538 (6)
C6—N1	1.341 (3)	Cu1—N2	2.0021 (17)
C7—O1	1.421 (3)	Cu1—N1	2.0092 (18)
C7—C8	1.499 (3)	Cu1—O1	2.0813 (14)
C7—H7A	0.9700		
			
O1—C1—C6	106.14 (17)	N2—C9—H9	118.9
O1—C1—H1A	110.5	C12—C9—H9	118.9
C6—C1—H1A	110.5	C12—C10—C11	118.8 (2)
O1—C1—H1B	110.5	C12—C10—H10	120.6
C6—C1—H1B	110.5	C11—C10—H10	120.6
H1A—C1—H1B	108.7	C10—C11—C8	119.4 (2)
N1—C2—C3	123.0 (2)	C10—C11—H11	120.3
N1—C2—H2	118.5	C8—C11—H11	120.3
C3—C2—H2	118.5	C10—C12—C9	119.3 (2)
C2—C3—C4	118.8 (3)	C10—C12—H12	120.4
C2—C3—H3	120.6	C9—C12—H12	120.4
C4—C3—H3	120.6	N2—Cu1—N1	155.06 (7)
C3—C4—C5	118.8 (2)	N2—Cu1—O1	78.29 (6)
C3—C4—H4	120.6	N1—Cu1—O1	77.99 (7)
C5—C4—H4	120.6	N2—Cu1—Cl2	98.17 (5)
C4—C5—C6	119.6 (2)	N1—Cu1—Cl2	97.71 (6)
C4—C5—H5	120.2	O1—Cu1—Cl2	147.65 (4)
C6—C5—H5	120.2	N2—Cu1—Cl1	93.36 (5)
N1—C6—C5	121.6 (2)	N1—Cu1—Cl1	96.97 (6)
N1—C6—C1	116.70 (18)	O1—Cu1—Cl1	96.81 (4)
C5—C6—C1	121.7 (2)	Cl2—Cu1—Cl1	115.53 (2)
O1—C7—C8	107.73 (16)	C2—N1—C6	118.2 (2)
O1—C7—H7A	110.2	C2—N1—Cu1	126.14 (16)
C8—C7—H7A	110.2	C6—N1—Cu1	115.53 (15)
O1—C7—H7B	110.2	C8—N2—C9	118.52 (18)
C8—C7—H7B	110.2	C8—N2—Cu1	115.92 (14)
H7A—C7—H7B	108.5	C9—N2—Cu1	125.45 (14)
N2—C8—C11	121.7 (2)	C7—O1—C1	115.70 (16)
N2—C8—C7	116.77 (18)	C7—O1—Cu1	112.11 (12)
C11—C8—C7	121.47 (19)	C1—O1—Cu1	111.27 (12)
N2—C9—C12	122.3 (2)		
			
N1—C2—C3—C4	0.7 (4)	Cl1—Cu1—N1—C6	81.62 (15)
C2—C3—C4—C5	0.2 (4)	C11—C8—N2—C9	−0.2 (3)
C3—C4—C5—C6	−1.0 (4)	C7—C8—N2—C9	−178.63 (18)
C4—C5—C6—N1	1.0 (4)	C11—C8—N2—Cu1	176.38 (15)
C4—C5—C6—C1	−179.2 (2)	C7—C8—N2—Cu1	−2.1 (2)
O1—C1—C6—N1	27.4 (3)	C12—C9—N2—C8	1.0 (3)
O1—C1—C6—C5	−152.4 (2)	C12—C9—N2—Cu1	−175.19 (16)
O1—C7—C8—N2	−20.0 (2)	N1—Cu1—N2—C8	33.8 (2)
O1—C7—C8—C11	161.56 (18)	O1—Cu1—N2—C8	15.52 (14)
C12—C10—C11—C8	0.7 (3)	Cl2—Cu1—N2—C8	162.86 (13)
N2—C8—C11—C10	−0.7 (3)	Cl1—Cu1—N2—C8	−80.74 (14)
C7—C8—C11—C10	177.7 (2)	N1—Cu1—N2—C9	−149.93 (18)
C11—C10—C12—C9	0.1 (3)	O1—Cu1—N2—C9	−168.19 (18)
N2—C9—C12—C10	−1.0 (3)	Cl2—Cu1—N2—C9	−20.85 (17)
C3—C2—N1—C6	−0.7 (4)	Cl1—Cu1—N2—C9	95.55 (17)
C3—C2—N1—Cu1	−176.6 (2)	C8—C7—O1—C1	160.80 (17)
C5—C6—N1—C2	−0.1 (3)	C8—C7—O1—Cu1	31.76 (19)
C1—C6—N1—C2	−180.0 (2)	C6—C1—O1—C7	−166.95 (17)
C5—C6—N1—Cu1	176.21 (18)	C6—C1—O1—Cu1	−37.49 (19)
C1—C6—N1—Cu1	−3.6 (2)	N2—Cu1—O1—C7	−26.88 (13)
N2—Cu1—N1—C2	143.8 (2)	N1—Cu1—O1—C7	160.88 (14)
O1—Cu1—N1—C2	162.1 (2)	Cl2—Cu1—O1—C7	−113.67 (13)
Cl2—Cu1—N1—C2	14.7 (2)	Cl1—Cu1—O1—C7	65.17 (13)
Cl1—Cu1—N1—C2	−102.4 (2)	N2—Cu1—O1—C1	−158.20 (14)
N2—Cu1—N1—C6	−32.2 (3)	N1—Cu1—O1—C1	29.56 (14)
O1—Cu1—N1—C6	−13.90 (15)	Cl2—Cu1—O1—C1	115.01 (13)
Cl2—Cu1—N1—C6	−161.33 (15)	Cl1—Cu1—O1—C1	−66.16 (13)
